# Structural Integrity and Cellular Viability of Cryopreserved Allograft Heart Valves in Right Ventricular Outflow Tract Reconstruction: Correlation of Histopathological Changes with Donor Characteristics and Preservation Times

**DOI:** 10.21470/1678-9741-2020-0710

**Published:** 2022

**Authors:** Ondrej Fabian, Mariia Havova, Roman Gebauer, Rudolf Poruban, Jaroslav Spatenka, Jan Burkert, Vilem Rohn, Vaclav Chaloupecky, Arnost Komarek, Tomas Kala, Jan Janousek

**Affiliations:** 1 Clinical and Transplant Pathology Centre, Institute for Clinical and Experimental Medicine, Praha, Czech Republic.; 2 Department of Pathology and Molecular Medicine, 3^rd^ Faculty of Medicine, Charles University and Thomayer Hospital, Praha, Czech Republic.; 3 Department of Cardiovascular Surgery, 2^nd^ Faculty of Medicine, Charles University and Motol University Hospital, Praha, Czech Republic.; 4 Children’s Heart Centre, 2^nd^ Faculty of Medicine, Charles University and Motol University Hospital, Praha, Czech Republic.; 5 Department of Transplantation and Tissue Bank, National Allograft Heart Valve Bank, 2^nd^ Faculty of Medicine, Charles University and Motol University Hospital, Praha, Czech Republic.; 6 Department of Probability and Mathematical Statistics, Faculty of Mathematics and Physics, Charles University, Praha, Czech Republic.

**Keywords:** Heart Valve, Allografts, Histopathology, Antigen Presenting Cells, Cryopreservation, Degeneration, Tissue Donors

## Abstract

**Introduction:**

Cryopreserved allograft heart valves (CAHV) show longer event-free survival compared to other types of protheses. However, all patients develop early and/or late allograft failure. Negative predictors are clinical, and there is a lack of evidence whether they correspond with the microscopic structure of CAHV. We assessed histopathological signs of structural degeneration, degree of cellular preservation, and presence of antigen-presenting cells (APC) in CAHV and correlated the changes with donor clinical characteristics, cryopreservation times, and CAHV types and diameters.

**Methods:**

Fifty-seven CAHV (48 pulmonary, nine aortic) used for transplantation between November/2017 and May/2019 were included. Donor variables were age, gender, blood group, height, weight, and body surface area (BSA). Types and diameters of CAHV, cold ischemia time, period from decontamination to cryopreservation, and cryopreservation time were recorded. During surgery, arterial wall (n=56) and valvar cusp (n=20) samples were obtained from the CAHV and subjected to microscopy. Microscopic structure was assessed using basic staining methods and immunohistochemistry (IHC).

**Results:**

Most of the samples showed signs of degeneration, usually of mild degree, and markedly reduced cellular preservation, more pronounced in aortic CAHV, correlating with arterial APC counts in both basic staining and IHC. There was also a correlation between the degree of degeneration of arterial samples and age, height, weight, and BSA of the donors. These findings were independent of preservation times.

**Conclusion:**

CAHV show markedly reduced cellular preservation negatively correlating with the numbers of APC. More preserved CAHV may be therefore prone to stronger immune rejection.

**Table:** 

Abbreviations, acronyms & symbols	
APC	= Antigen-presenting cells
BSA	= Body surface area
CAHV	= Cryopreserved allograft heart valves
ELA	= Elastic fiber
HE	= Hematoxylin and eosin
IHC	= Immunohistochemistry
IQR	= Interquartile range
MEMA	= Mucoid extracellular matrix accumulations

## INTRODUCTION

Several types of prosthetic (mechanical and biological) heart valves had been used for restoration of continuity between right heart ventricle and pulmonary arteries, and most of them was abandoned due to unsatisfactory long-term results^[1]^. As the quality of the preservation techniques improved, cadaveric cryopreserved allograft heart valves (CAHV) slowly became conduits of choice, showing better hemodynamic performance, higher resistance to infections, lower risk of thromboembolic events, technical ease of transplantation, and longer event-free survival compared to other types of biological and mechanical prostheses^[2-4]^. However, despite the unequivocal advantages, all patients develop early or late allograft failure or dysfunction. The well documented negative clinical predictors are young age of donor, young age of recipient, low weight of recipient, small size of allograft, aortic type of allograft, and longer period of warm ischemia^[3-7]^. However, there is a lack of evidence whether any of these clinical predictors reflect in the microscopic structure of CAHV.

In this prospective study, we assessed histopathological changes in CAHV. The goals of this study were to evaluate signs of structural degeneration and the degree of cellular preservation and highlight the presence of immunogenic antigen-presenting cells (APC). Also, we aimed at the correlation of the given microscopic changes with donor clinical characteristics, cryopreservation times, and types and diameters of CAHV.

## METHODS

### Allograft Heart Valve Procurement and Preparation

In the Czech National Program of the Tissue and Organ Harvesting, all donor hearts not suitable for transplantation as a whole organ are harvested for CAHV processing. The age limit for the donor is usually set from matured newborn up to 65 years. Allografts are taken during standard multi-organ harvesting or from non-heart beating donors. Immediately after the heart harvest, a cold perfusion is initiated, and the heart is placed into a container with ice slush. The time period in the slush is referred to as the cold ischemia time, and a period up to 48 hours is accepted. In an “ideal” heart harvest from beating heart donor, the warm ischemia is avoided. After that, the hearts are dissected under sterile conditions. The status of the valve is assessed by a cardiac surgeon who excludes any macroscopically visible morphological alterations and tests the valve competency by perfusion with Ringer’s solution. Apart from that, samples of aortic and pulmonary trunk wall from peripheral margins of the conduits are taken and sent with transversely sectioned heart lamella to a pathologist for microscopic evaluation as a part of standard biopsy screening. The suitable allograft is decontaminated in a solution containing combination of antibiotics. It is usually stored for 24 hours in room temperature and then placed in a fridge for several days, with an upper limit of 28 days. Thorough quality and safety testing take place during this period. After that, the allograft is frozen in liquid nitrogen and stored in a tissue bank. A shelf life was arbitrary set at five years^[8,9]^. According to the National Allograft Heart Bank own validation, the expiration was recently extended up to six years for aortic and up to eight years for pulmonary cryopreserved allografts^[10]^. The final decision concerning the release of a particular allograft into the inventory for distribution is done by a person responsible for the tissue establishment. Then, the decision concerns all donors based on the available tissue data, starting from donor anamnestic records, obligatory serological testing, and microbiological screening, partial heart autopsy report, and biopsy report^[11]^. Thawing of the allograft before its implantation is gradual. After its removal from the container, the allograft is left in room temperature for 15 minutes. After that, it is placed in 37-40°C water bath for at least 15 minutes.

### Gathering Clinical Data and Tissue Sampling

Fifty-seven CAHV (48 pulmonary, nine aortic) distributed by the National Allograft Heart Valve Bank and subsequently used for right ventricular outflow tract reconstruction in the period between November 2017 and May 2019 were included in this prospective study. The donors’ clinical characteristics were recorded. Specific variables included age, gender, blood group, height, weight, and body surface area calculated in square meters using the Mosteller formula^[12]^. Also, the type of the allograft, its diameters, duration of cold ischemia, period from the end of decontamination to initiation of cryopreservation, and cryopreservation time were included in the subsequent analysis. Clinical details of the donors, allograft characteristics, and cryopreservation data are provided in the Table 1.

**Table 1:** Clinical details of the cadaveric donors and allograft valves’ characteristics.

Donor gender, male (%)		26 (46)
Donor age, years, median (mean, IQR)		36 (33, 13-53)
Height, centimeters, median (mean, IQR)		165 (156, 150-175)
Weight, kilograms, median (mean, IQR)		65 (64, 50-80)
BSA, square meters, median (mean, IQR)		1.75 (1.62, 1.45-1.97)
Blood group, ABO/Rh system (n)		A+ (18), A- (3), B+ (6), B- (1), AB+ (5), AB- (1), O+ (19), O- (4)
Allograft type (n)		Pulmonary (48), aortic (9)
Allograft diameter, millimeters, median (mean, IQR)	Pulmonary	25 (24, 23-26)
	Aortic	14 (14, 12-16)
Allograft length, millimeters, median (mean, IQR)	Pulmonary	40 (41, 33-50)
	Aortic	60 (57, 50-65)
Duration of cold ischemia, minutes, median (mean, IQR)		995 (1000, 733-1281)
Period from the end of decontamination to initiation of cryopreservation, days, median (mean, IQR)		16 (16, 12-21)
Cryopreservation time, days, median (mean, IQR)		150 (302, 84-352)

BSA=body surface area; IQR=interquartile range

During the right ventricular outflow tract reconstruction, a full-thickness arterial wall sample was obtained from the CAHV. In cases in which the transplantation was preceded by reduction of the semilunar valve size, a sample of the CAHV’s valvar cusp was taken as well. Bifurcations were avoided during the arterial wall sampling because slight microscopic changes may be physiologically seen in this region, and they could falsely overestimate the assessment of the structural degeneration. At the end, 56 arterial wall samples (47 pulmonary, nine aortic) and 20 valve cusp samples (17 pulmonary, three aortic) were subjected to histopathological evaluation by light microscopy. All the microscopic slides were evaluated by a senior cardiovascular pathologist.

### Histopathological Assessment

Each specimen was fixed in 10% buffered formalin, embedded in paraffin block, and sections of 2 µm thickness were taken. All the microscopic slides were stained with hematoxylin and eosin (HE). To properly assess the microscopic signs of structural degeneration, additional stains highlighting individual components of the arterial wall and valvar cusp were performed. All samples were stained with Masson’s trichrome highlighting smooth muscle cells and fibrous tissue, Weigert’s resorcin fuchsin highlighting elastic fibers, and Alcian blue/periodic acid-Shiff stain demonstrating presence of mucoid extracellular matrix accumulations (MEMA). The specific morphological variables assessed in the arterial wall included elastic fiber fragmentation/loss, elastic fiber thinning, elastic fiber disorganization, presence of laminar medial necrosis, intralamellar and translamellar MEMA, fibrosis, neovascularization, calcification, necrosis, and presence of atherosclerotic intimal plaque. Standard definitions of the given variables corresponded with the international consensus statement for degenerative aortic diseases from the Society of Cardiovascular Pathology and the Association for European Cardiovascular Pathology^[13]^. In the valvar cusp, the assessed variables included elastic fiber reduction, myxoid change, fibrosis, calcifications, hemorrhages, necrosis, and overall differentiability of the individual valve layers (zona fibrosa, spongiosa, and ventricularis). The assessment of preservation of cellular component was based on the standard HE stain and immunohistochemistry. The HE assessment relied on the degree of nuclear pyknosis, cellular shrinkage, and cellular loss. For the immunohistochemistry, 1-µm tissue sections were deparaffinized, and the specific primary antibodies were used: anti-vimentin (DAKO, at dilution 1:100) highlighting the overall cellularity of the sample, anti-h-caldesmon (BioSB, at dilution 1:100) demonstrating smooth muscle cells, and anti-CD34 (BioGenex, at dilution 1:40) staining endothelial cells. Most of the aforementioned variables were graded 0 to 3 (absent, mild, moderate, severe), except for fibrosis, calcification, necrosis, hemorrhage, neovascularization, and atherosclerosis, which were graded 0 or 1 (absent or present). To highlight a presence of immunogenic cells, immunohistochemical staining of S100β (CellMarque, at dilution 1:300), CD20 (DAKO, at dilution 1:300), CD3 (BioGenex, at dilution 1:100), and CD8 (DAKO, at dilution 1:200) antigens was performed and the number of positive cells for 10 high-power fields (×400) was counted. Anti-S100β was used to highlight APC, CD20 for B-lymphocytes, and CD4 and CD8 for corresponding subtypes of T-lymphocytes. The presence of neutrophils and eosinophils in HE was evaluated as well. HRP/DAB PolyDetector (Bio SB) was used as a detection kit for all the aforementioned antibodies.

### Ethical Considerations

Legal representatives of the patients signed an informed consent form for inclusion into the study. The study was approved by the Ethics Committee of Motol University Hospital (reference number EK-930/18).

### Statistical Analysis

A programming language Python (version 3.7.7) with SciPy library (version 1.4.1) was used for data analysis. All the variables included in the study were pairwise correlated. The histopathological variables were also assessed as groups by the mean of a canonical correlation analysis. The groups of variables represented arterial and valvar structural degeneration and cellular viability. To evaluate pairwise associations, Pearson’s correlation coefficient was used. To assess the independence within pairs of variables, the following statistical tests were applied: test on correlation coefficient for numeric clinical and numeric histopathological data, chi-squared test of independence for categorical clinical and nominal histopathological data, Welch’s t-test for binary clinical and numeric histopathological data, and Welch’s analysis of variance (or ANOVA) for categorical clinical and numeric histopathological data. P-values of < 0.05 were considered significant. A 95% confidence interval was used.

## RESULTS

### Structural Degeneration

**Fig. 1:**
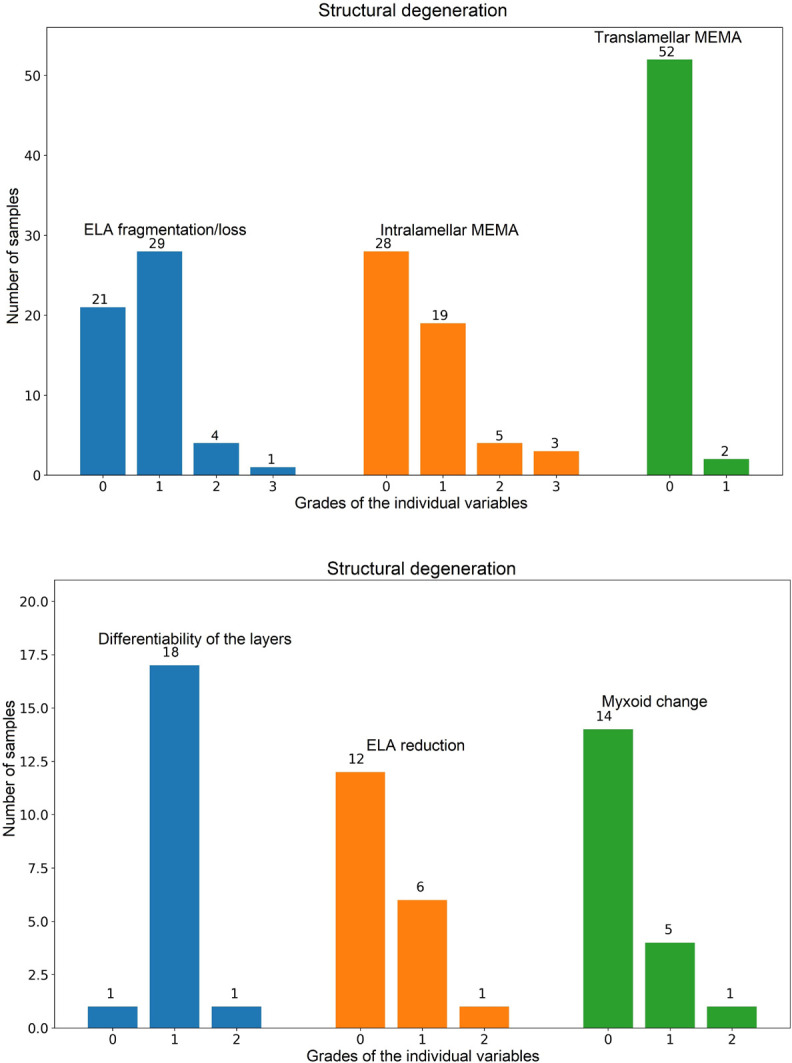
Bar plots showing individual components of structural degeneration in arterial (A) and valvar (B) samples (only the non-zero variables are shown). X-axis shows grades of severity for each individual variable, y-axis provides number of samples in each category (different colors of the bars were used only for a better understandability of the picture). ELA=elastic fiber; MEMA=mucoid extracellular matrix accumulations.

Thirty-four arterial samples showed signs of elastic fiber fragmentation/loss, graded as mild (grade 1) in 29 cases, moderate (grade 2) in four cases, and severe (grade 3) in one case. Intralamellar MEMA were present in 27 cases, from which 19 were graded as mild, five as moderate, and three cases as severe. Translamellar MEMA were found only in two cases, and both were graded as mild. Among the valvar samples, 18 cases showed mildly reduced and one case moderately reduced overall differentiability of the individual layers. Six samples harbored mild elastic fiber reduction, and one case moderate reduction. Myxoid changes were present in six cases, from which five were graded as mild, and one case as moderate (Figure 1). Not a single arterial or valvar sample showed elastic fiber thinning, elastic fiber disorganization, laminar medial collapse, fibrosis, calcification, neovascularization, necrosis, or hemorrhage.

### Cellular Preservation

**Fig. 2:**
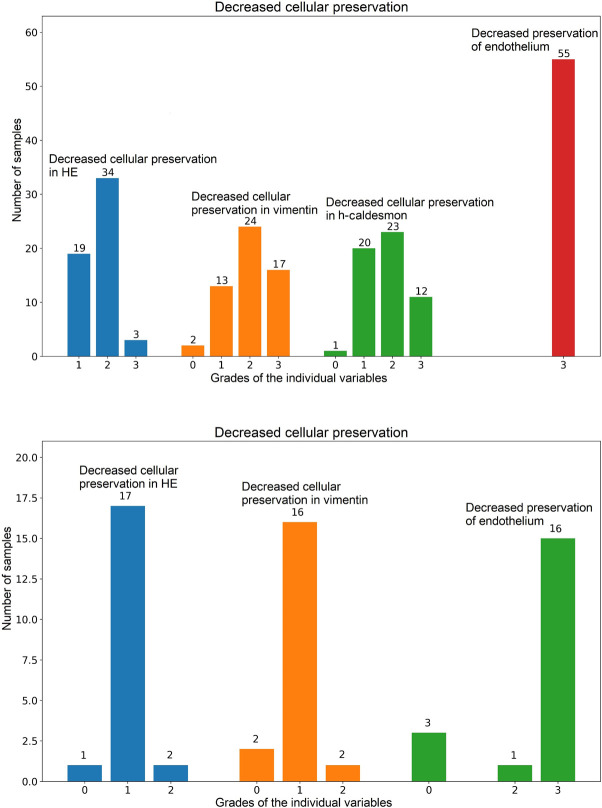
Bar plots showing individual components of decreased cellular viability in arterial (A) and valvar (B) samples (only the non-zero variables are shown). X-axis shows grades of severity for each individual variable, y-axis provides number of samples in each category (different colors of the bars were used only for a better understandability of the picture). HE=hematoxylin and eosin.

All arterial samples showed some level of decreased cellular preservation based on the HE findings. Nineteen cases were graded as mild, 34 cases as moderate, and three cases as severe. Vimentin stain showed mildly decreased cellular preservation in 13 cases, moderate in 24 cases, and severe in 17 cases. All samples except for one harbored decreased preservation of smooth muscle cells in h-caldesmon stain, graded as mild in 20 cases, moderate in 23 cases, and severe in 12 cases. All samples showed complete (grade 3) loss of endothelial lining highlighted by CD34 stain. Regarding the valves, all samples except for one showed decreased cellular preservation in HE. Seventeen cases were graded as mild, and two cases as moderate. In vimentin stain, 16 cases showed mild loss of cellular preservation, and two cases moderate. Sixteen cases had complete loss, and one case moderate loss of endothelial lining. In three cases the endothelium was preserved (Figure 2).

### Immunogenic Cell Counts

A mean number of five S100β+ cells (median one cell, interquartile range 0-5 cells) was counted for the arterial samples. In case of valvar samples, a mean count was 41 cells (median 37 cells, interquartile range 27-54 cells). No CD20, CD3, or CD8+ lymphocytes nor eosinophils or neutrophils were found.

**Fig. 3:**
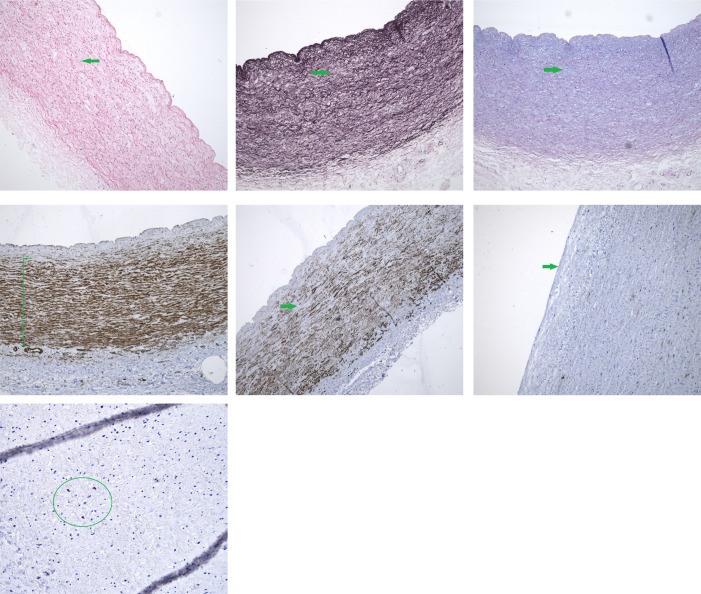
Photomicrographs of pulmonary trunk wall. A) Mild reduction of cellular preservation showing shrunken and pyknotic nuclei (arrow), hematoxylin and eosin stain, magnification 100×; B) mild fragmentation and loss of elastic fibers (arrow), Weigert’s resorcin fuchsin stain, magnification 100×; C) mild focal intralamellar mucoid extracellular matrix accumulations (arrow), Alcian blue/periodic acid-Schiff stain, magnification 100×; D) anti-vimentin immunohistochemical stain demonstrating well preserved mesenchymal cells (bar), magnification 100×; E) anti-h-caldesmon immunohistochemical stain demonstrating mild focal loss of smooth muscle cells (arrow), magnification 100×; F) anti-CD34 immunohistochemical stain demonstrating complete loss of endothelial cells (arrow), magnification 200×; G) anti-S100β immunohistochemical stain demonstrating a few positive antigen-presenting dendritic cells (circle), magnification 400×.

**Fig. 4:**
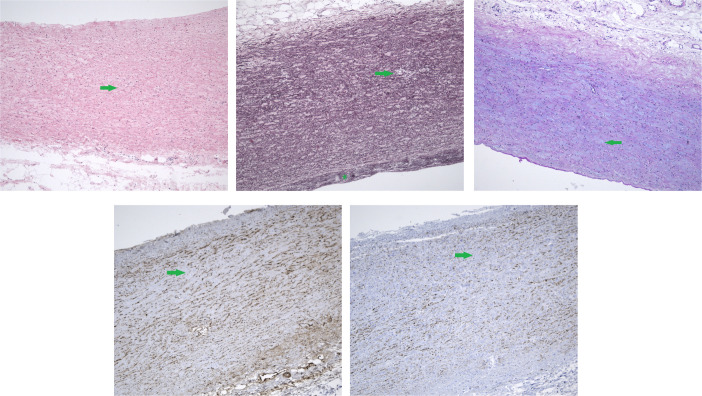
Photomicrographs of aortic wall. A) Marked reduction of cellular preservation showing shrunken and pyknotic nuclei (arrow), hematoxylin and eosin stain, magnification 100×; B) mild fragmentation elastic fibers (arrow) and mild intimal fibrosis (asterisk), Weigert’s resorcin fuchsin stain, magnification 100×; C) mild intralamellar mucoid extracellular matrix accumulations (arrow), Alcian blue/periodic acid-Schiff stain, magnification 100×; D) anti-vimentin immunohistochemical stain demonstrating marked reduction of mesenchymal cells (arrow), magnification 200×; E) anti-h-caldesmon immunohistochemical stain demonstrating marked reduction of smooth muscle cells (arrow), magnification 200×.

Representative microphotographs demonstrating given histopathological changes in aortic and pulmonary arterial wall and valvar cusp samples are shown in Figures 3 and 4.

### Comparative Analysis of Microscopic Findings

In case of arterial samples, a statistically significant correlation was found between the degree of elastic fiber fragmentation/loss and decreased cellular preservation in HE (r=0.3367; *P*=0.0053). In case of valvar cusps, there was a correlation between reduced differentiability of the individual layers and decreased preservation in both HE (r=0.4117; *P*=0.0005) and vimentin stain (r=0.3536; *P*=0.0467). The other correlations comparing individual pairs of variables were not significant, as well as canonical correlations between grouped variables representing overall structural degeneration and cellular preservation.

Regarding the immunogenic cell counts, a significant negative correlation was found between arterial S100β+ cell counts and degree of elastic fiber fragmentation/loss (r=-0.4016; *P*=0.0038), decreased cellular preservation in HE (r=-0.5212; *P*=0.0001), and degrees of overall structural degeneration (r=-0.4063; *P*=0.0455) and overall cellular preservation as grouped variables (r=-0.5811; *P*=0.0004). No significant correlation was found for valvar cusps.

### Correlation of Histopathological Changes with Clinical Variables

Since the high number of individual variables included in the study posed a risk of accidental correlation, which would not necessarily imply a direct causation, only the grouped histopathological variables were subsequently used in this sub-analysis. A significant correlation was found between the degree of structural degeneration of arterial samples and age (r=0.6033; *P*<0.0000), height (r=0.4288; *P*=0.0137), weight (r=0.5139; *P*=0.0011), and body surface area (r=0.4965; *P*=0.0019) of the donors. No correlation was found for valvar cusps.

Neither arterial nor valvar samples showed any significant correlation between the degree of structural degeneration and duration of cold ischemia, period of decontamination, and cryopreservation time. Also, no correlation was revealed between clinical variables and a degree of cellular preservation or S100β+ cell counts.

Arterial samples from aortic allografts showed more extensive loss of cellular preservation in vimentin (*P*=0.0066) and h-caldesmon (*P*<0.0000) stains compared to pulmonary allografts. However, there was no significant difference in S100β+ cell counts between arterial and pulmonary allografts. No association was found between any clinical variable and homograft type.

## DISCUSSION

Better hemodynamics and durability ensure a superior longevity of CAHV compared to other types of valved conduits such as xenografts or mechanical prostheses. Negative predictors of early or late failure stem from clinical studies include young age of donor, small size of allograft, young age of recipient, low weight of recipient, aortic type of allograft, and duration of warm ischemia^[3-7]^. The first two aforementioned predictors are probably related to the minimal to naught growth capacity of the allograft^[4]^. Young age of the recipient may affect the allograft survival also indirectly, since small children often undergo surgery for complex heart malformations with uncertain prognosis^[4]^. Warm ischemia causes damage to the allograft and is usually followed by reparative process with fibroproduction^[4]^ (all of our CAHV in the study were harvested without any warm ischemia). However, there is a lack of data concerning whether any of these clinical variables correspond with specific microscopic findings. An eventual predictive value of histopathological examination of cryopreserved allografts prior their implantation thus remains unclear.

In our study, we assessed the presence of structural degeneration, decreased cellular preservation, and preserved immunogenic cells and correlated the given changes with clinical variables. The signs of structural degeneration were generally mild and may have already been present at the time of harvest. However, a possible effect of cryopreservation, temporary ischemia, or handling cannot be excluded, since the light microscopy may not be sensitive enough to detect more subtle changes^[14]^. In the study, we failed to prove any correlation between the degree of microscopic degeneration and the duration of cold ischemia, period from the end of decontamination to initiation of cryopreservation, or cryopreservation time. These findings concur with certain previous studies. Fiala et al.^[8]^ did not documented any changes in the amount of collagen and elastin or any changes in response to tensile stress in cryopreserved allografts after 10 years of preservation. Kubikova et al.^[15]^ did not find any correlation between microscopic structure of aortic and pulmonary CAHV and their mechanical properties. However, some studies examining allograft ultrastructure^[16]^ show substantial structural deterioration of the collagen on the ultrastructural level compared to fresh allografts. CAHV may also show changes in extracellular matrix on functional level. Based on a study by Kano et al.^[17]^, a collagen metabolism seems to be altered towards the degradable side, with relatively maintained collagen synthesis, but decreased overall protein synthesis and increased activity of collagenolysis.

On the other hand, the negative effect of the cryopreservation on the cellular component of the allografts is evident. All the arterial samples in our study and all valvar samples except for one showed signs of decreased cellular preservation, usually of higher degree. It is clear that the viability of the cell cannot be based only on the nucleus patterns in HE and immunohistochemical detection of cellular antigens, such as vimentin or h-caldesmon. These methods are not a definitive proof that the cell is dead. However, we can certainly confirm a significant degree of cellular regression. Nevertheless, there was no correlation of the degree of the devitalization with the preservation times. Temporary ischemia, damage due to handling, or reperfusion injury after the implantation may also lead to variable loss of viable cells^[14,18]^. The endothelium seems to be the most vulnerable structure. Previous studies declare high sensitivity of the endothelia to ischemia. When stored in antibiotic solution, endothelial cells start to lose viability after 24 hours, and the time may vary based on the length of the warm and cold ischemia or the donor comorbidities^[6]^. In case of CAHV from our department, almost complete loss of the endothelial layer and disruption of the basement membrane obviously occur during the harvesting already by exposure to the saline^[19,20]^. Our current study supports these findings, since the endothelium was markedly denuded in the vast majority of the samples. We also demonstrated a significant correlation between degrees of structural degeneration and decreased cellular preservation in both arterial and valvar samples. The interpretation of these results is challenging since there is a lack of other studies supporting our findings. Therefore, we can only speculate that disrupted extracellular matrix may have enhanced detachment and subsequent loss of cells during allograft preservation.

A degree of cellular preservation of the allograft is reflected by a degree of preserved immunogenicity^[6]^. Earlier studies emphasized on the preservation of cellular viability, since the viable fibroblasts may ensure a better durability of the allograft^[21]^. However, a preserved cellular viability is accompanied by viable immunogenic cells, which may play a role in development of an early or late allograft failure. It is necessary to stress that a degree of contribution of immunological reaction to allograft failure still remains unclear^[22]^. However, some sort of immunologically driven mechanism towards the allograft is undoubted. Such immune response may be accentuated especially in small children. Many patients who underwent CAHV transplantation subsequently developed serum positivity of anti-human leukocyte antibodies against the donor tissue^[6,14]^. In our study, we aimed at the assessment of viable S100β+ APC together with basic lymphocyte counts. Obviously, counting of APC is far from global approach to elucidate complex mechanisms of allograft immunogenicity. However, according to previous studies, viable APC seem to be key players^[14]^. Our results demonstrated a significant negative correlation between the level of decreased cellular preservation and numbers of S100β+ cells. Based on our results, allografts with better cellular preservation may be prone to stronger immune response. Some authors even recommend a temporary perioperative immunosuppression^[5]^. However, we failed to prove any association between S100β+ cell counts and the duration of cold ischemia, period from the end of decontamination to initiation of cryopreservation, or cryopreservation time. Apart from APC, a viable endothelium seems to play a role in the allograft immunogenicity as well. According to previous studies^[23,24]^, viable endothelial cells show high level of antigenicity. On the other hand, decreased cellular preservation may negatively affect durability of the allograft, if combined with a higher degree of structural degeneration. Given the fact that restitution of extracellular matrix requires viable interstitial cells^[14]^, using a markedly degenerated allograft in combination with its decellularization may have a negative impact on its longevity. Disrupted extracellular matrix itself may also serve as a nucleation center for calcification^[14]^. The aortic allografts showed significantly higher degree of devitalization compared to the pulmonary type, which might indicate their eventual lower immunogenicity. However, several studies^[4,5]^ considered the aortic type of allograft as one of the predictors of decreased longevity. A possible explanation might be a different pathogenesis of an early and late allograft failure. Baskett RJ et al.^[6]^ suggested that frequent occurrence of early allograft dysfunction between first six to 12 months after the surgery in pediatric patients is most likely immune-mediated. A long-term failure, on the other hand, may be a consequence of non-immune degenerative process, probably caused by higher intrinsic amount of elastin and calcium in aortic allografts, making them more susceptible for subsequent calcification^[25]^. At any rate, a possible higher sensitivity of pulmonary allografts to the aforementioned immune-mediated early failure cannot be fully confirmed, since we did not find any statistically significant difference in preserved S100β+ immune cell counts compared to the aortic allografts.

A continuous effort to decrease antigenicity led to the introduction of decellularized allografts. The mechanism of decellularization is based on withdrawal of all viable cells from the extracellular matrix while the structural integrity of the allograft is preserved. The original tissue is subsequently replaced by migration of recipient-specific cells. Methods of decellularization are various, and they have been applied to both fresh and cryopreserved allografts. Very promising and impressive results of other authors led many surgeons and cardiologists to consider the allograft decellularization as a way to decreased its immunogenicity and increase its longevity, especially in children^[26]^. Considering our findings and results of previous studies, we could speculate that children that are prone to stronger immune response could benefit from decellularized allografts from younger donors with well-preserved microscopic structure. On the other hand, the decellularization may have not been an appropriate technique for allografts from older donors with more advanced structural deterioration. As mentioned above, such allografts combined with decellularization may pose an increased risk of accelerated degeneration. However, this hypothesis needs to be confirmed by other prospective studies with long-term follow-up of the recipients.

### Limitations

This study has several limitations. The number of valvar cusp samples included in this study was limited, and most of the significant findings were thus related to the arterial samples. Since CAHV were subsequently used for transplantation, valvar fragments could have been sampled only in case of reduction of the valve. Apart from that, the histopathological signs of structural degeneration were often very subtle and may have been prone to intrapersonal variability. Due to their delicacy, we also could not conclude with certainty if they were really caused by cryopreservation, temporary ischemia, or handling. Also, the aforementioned international consensus for the assessment of degenerative changes was primarily established for aortas, whereas the microscopic structure of the pulmonary trunk is slightly different. However, the difference is quantitative and not qualitative, thus the official criteria can be applied for pulmonary allografts as well. In addition, all the changes were assessed by a senior cardiovascular pathologist, and we strongly believe in reproducibility and objectiveness of this pulmonary CAHV assessment.

## CONCLUSION

In conclusion, microscopic structural alterations can be detected in almost all cryopreserved allografts and affect both arterial wall and free valvar cusps. They were usually mild and, in case of arterial wall, correlated with age, height, weight, and body surface area of donors. A cellular preservation of the allografts was markedly decreased and negatively correlated with the numbers of preserved APC. These findings were independent of the donor characteristics and cryopreservation times. Additional studies with long-term prospective follow-up of the recipients need to be performed to confirm the predictive value of the given histopathological changes for early or late allograft failure.
